# The RNA binding protein hnRNP-K mediates post-transcriptional regulation of uncoupling protein-2 by angiopoietin-1

**DOI:** 10.1016/j.cellsig.2014.03.005

**Published:** 2014-07

**Authors:** Tariq A. Tahir, Harprit Singh, Nicholas P.J. Brindle

**Affiliations:** Departments of Cardiovascular Sciences and Biochemistry, University of Leicester, Henry Wellcome Building, Lancaster Road, Leicester LE1 9HN, UK

**Keywords:** Endothelial, Angiopoietin, hnRNP-K, Uncoupling protein-2, Reactive oxygen species

## Abstract

Angiopoietin-1 (Ang1) is a ligand for the receptor tyrosine kinase Tie2 and has key roles in the development of the vascular system and vascular protection. In a screen to define signalling pathways regulated by Ang1 in endothelial cells we found the RNA-binding protein hnRNP-K to be phosphorylated in response to Ang1. The ligand stimulated both tyrosine phosphorylation of hnRNP-K and recruitment of the tyrosine kinase Src to the RNA-binding protein. In endothelial cells hnRNP-K was found bound to mRNA encoding the mitochondrial protein uncoupling protein-2 (UCP2). Ang1 stimulation of cells resulted in the release of UCP2 mRNA from hnRNP-K. Using *in vitro* assays we confirmed direct binding between hnRNP-K and UCP2 mRNA. Furthermore Src induced phosphorylation of purified hnRNP-K and prevented UCP2 mRNA binding. Tyrosine 458 in the RNA-binding protein was found to be required for suppression of UCP2 mRNA binding by Src phosphorylation. In addition to releasing UCP2 mRNA from hnRNP-K, Ang1 induced an increase in UCP2 protein expression in endothelial cells without affecting total UCP2 mRNA levels. Consistent with the known effects of UCP2 to suppress generation of reactive oxygen species, Ang1 limited ROS production in endothelium stimulated with tumour necrosis factor-α. Taken together these data suggest that UCP2 mRNA is present in endothelial cells bound to hnRNP-K, which holds it in a translationally inactive state, and that Ang1 stimulates Src interaction with hnRNP-K, phosphorylation of the RNA-binding protein, release of these transcripts and upregulation of UCP2 protein expression. This study demonstrates a new mechanism for post-transcriptional regulation of UCP2 by the vascular protective ligand Ang1. The ability to rapidly upregulate UCP2 protein expression may be important in protecting endothelial cells from excessive generation of potentially damaging reactive oxygen species.

## Highlights

• In endothelial cells UCP2 mRNA is bound to hnRNP-K.
• hnRNP-K suppresses UCP2 mRNA translation.
• Ang1 stimulates recruitment of Src, phosphorylation of hnRNP-K and mRNA release.
• Src phosphorylation of tyrosine-458 in hnRNP-K suppresses UCP2 mRNA binding.
• Ang1 increases UCP2 protein levels.

## 1 Introduction

Angiopoietin-1 (Ang1) is a secreted ligand essential for development of the vascular system as well as maintenance and protection of the mature vasculature [1,2]. This ligand is a glycoprotein of approximately 70 kDa [3] and, together with angiopoietins 2, -3 and -4, is a member of the angiopoietin family of proteins [1,2]. In development Ang1 is required for the latter stages of blood vessel formation, where it acts to regulate branching and interaction of endothelial cells in nascent vessels with extracellular matrix and perivascular support cells [4,5]. Post-development effects of Ang1 are broadly protective and the ligand has been shown to inhibit microvessel regression, suppress vascular inflammation and inhibit vessel leakage [1,2,6]. The protective effects of Ang1 are important in maintaining a quiescent and stable vasculature [1]. At the cellular level Ang1 acts to suppress apoptosis, enhance migration, promote monolayer integrity and inhibit inflammatory gene expression in endothelial cells [1,2,6].

The primary receptor for Ang1, and the other angiopoietins, is the receptor tyrosine kinase Tie2 [3]. Binding of Ang1 to Tie2 results in phosphorylation and activation of the receptor and stimulation of downstream signalling cascades including the phosphatidylinositol-3-kinase pathway [7]. This pathway has key roles in the anti-apoptotic actions of Ang1 [8,9]. Phosphatidylinositol-3-kinase also contributes to Ang1 suppression of endothelial inflammation [10], as does ABIN2, an intermediate that is recruited to activated Tie2 and acts as an inhibitor of the inflammatory transcription factor NFkB [11,12]. Another signalling intermediate involved in Ang1 action is the protein Dok-2 [13]. This adaptor protein is recruited to the activated receptor where it gets phosphorylated leading to binding of Nck and p21-activating kinases that are involved in Ang1 activated endothelial migration [14].

Although a number of signalling pathways have been implicated in mediating Ang1 actions, our understanding of how this ligand regulates cellular function is still comparatively poor. In particular we know relatively little about the pathways involved in the cellular protective effects of Ang1. In order to uncover signalling components mediating Ang1 effects, therefore, we performed a proteomic screen to identify proteins that become tyrosine phosphorylated in response to Ang1 in endothelial cells. Here we report the RNA-binding protein heterogeneous nuclear ribonucleoprotein-K (hnRNP-K) as a new intermediate in Ang1 signalling involved in post-transcriptional control of the antioxidant mitochondrial uncoupling protein-2.

## 2 Materials and methods

### 2.1 Materials

Recombinant human Angiopoietin1 was purchased from R&D Systems and used at a concentration of 2.8 nM (monomer). UCP2 antibodies were from Biolegend, and hnRNP-K antibodies were from Abcam. All other reagents were as previously detailed [11]. hnRNP-K cDNA was a kind gift from Professor Sui Huang (Northwestern University Medical School, Chicago, IL, USA).

### 2.2 Cell culture

Human umbilical vein endothelial cells (HUVEC) were purchased from Promocell and maintained in medium 199 supplemented with 20% fetal calf serum (FCS), heparin 5 units/ml and endothelial cell growth supplement (50 μg/ml). Only passages 3–5 were used in experiments. The EA.hy.926 endothelial cell line [15] was a generous gift from C. J. Edgell (University of North Carolina) and were maintained Dulbecco's modified Eagle's medium supplemented with 10% FCS. Chinese Hamster Ovary (CHO) cells were maintained in minimum essential media supplemented with 10% FCS. All cells were cultured at 37 °C in a humid atmosphere containing 5% CO_2_ in air. For transfection, 80% confluent cells were transfected with plasmid DNA using lipofectamine 2000 according to manufacturer's protocol. Cells were cultured for 18 h post-transfection before lysis.

### 2.3 Immunoprecipitation and Immunoblotting

Before lysis cells were washed with PBS then lysis buffer (100 mM KCl, 5 mM MgCl_2_, 10 mM HEPES pH 7.0, and 0.5% Nonidet P-40 detergent, 1 mM DTT, protease inhibitor cocktail) added. The cells were scraped and the lysate centrifuged at 10,000 *g* for 10 min at 4 °C. For immunoprecipitation, relevant antibodies were added to the supernatants and incubated at 4 °C on a roller for 3 h after which protein-A sepharose was added and left for a further 3 h on the roller at 4 °C. The immunoprecipitate was centrifuged at 3000 *g* for 1 min and washed in lysis buffer 3 times. Proteins were eluted by the addition of Laemmli sample buffer containing 100 mM dithiothreitol and heated to 95 °C for 5 min before being resolved by SDS–PAGE. For the analysis of whole cell protein extracts, lysates were mixed with Laemmli buffer as above. Proteins were transferred to nitrocellulose membranes, probed with the relevant antibodies and visualized with peroxidase-conjugated secondary antibodies and chemiluminescent detection. Optical densities of bands on immunoblots were quantified using ImageJ software.

### 2.4 RNA co-immunoprecipitation

RNA was isolated from immunoprecipitates using the protocol of Tenenbaum [16] with a few modifications. Cells were lysed directly in lysis buffer (100 mM KCl, 5 mM MgCl_2_, 10 mM HEPES pH 7.6, and 0.5% Nonidet P-40 detergent, 1 mM DTT, 100 U/ml SuperaseIn RNase inhibitor and protease inhibitor cocktail). Cells were scraped and centrifuged at 14,000 *g* for 10 min at 4 °C. The supernatant was removed and re-centrifuged at 14,000 *g* for 10 min at 4 °C. Antibodies were allowed to bind to protein-A beads for 2 h at 4 °C in NT2 buffer (50 mM Tris pH 7.4, 150 mM NaCl, 1 mM MgCl2, 0.05% Nonidet P-40 before addition to the supernatant. The supernatant was added to antibody coated protein-A beads with the addition of 0.75 ml NT2 buffer, 1 mM DTT, 15 mM EDTA and an extra 100 U/ml of RNase inhibitor. The immunoprecipitation was allowed to proceed for 3 h at room temperature with agitation after which immunoprecipitates were centrifuged at 3000 *g* for 20 s. The beads were washed in NT2 buffer 3 times. The beads were then resuspended in NT2 buffer with the addition of 0.1% SDS and 30 μg of proteinase K and incubated at 55 °C for 30 min. Immunoprecipitated RNA was isolated using phenol:chloroform:isoamyl alcohol extraction and ethanol precipitation.

### 2.5 PCR

Isolated RNA was reverse transcribed using RETROscript (Ambion) and random decamer primers. The cDNA was used as template for subsequent PCR to screen for the presence of UCP2 using the primers UCP2 forward (5′ATGGTTGGGTTCAAGGCCACAGAT3′) and UCP2 reverse (5′CATGCTCAGAGCCCTTGGTGTAGA3′).

### 2.6 Subcloning, expression and purification of hnRNP-K

Full-length human hnRNP-K was cloned into the *Escherichia coli* expression vector pLEICS-01 (University of Leicester) encoding an N-terminus His6-tag using the primers; forward 5′TACTTCCAATCCATGGAAACTGAACAGCCAGAAGAAACCT3′ and reverse 5′TATCCACCTTTACTGTCATTAGAATCCTTCAACATCTGCA3′.

BL21 (Novagen) were transformed with hnRNP-K expression vector using the manufacturer's instructions. Transformed *E. coli* was grown in Luria-Bertani broth at 37 °C, 250 rpm to an optical density of 0.6 (600 nm) after which cells were induced for expression of hnRNP-K using Isopropyl β-D-1-thiogalactopyranoside at 1 mM at 25 °C, 250 rpm for 4 h. Cells were harvested and lysed using 10 ml of lysis buffer (50 mM Tris pH8.0, 150 mM NaCl, 5% glycerol, 10 mM imidazole pH 8.0, protease inhibitor cocktail from Roche). The cells were sonicated in the lysis buffer. The debris was pelleted at 15,000 g at 4 °C for 30 min. The supernatant was incubated with 1 ml of nickel beads (Ni-NTA superflow from Qiagen) for 16 at 4 °C under gentle rocking. The beads were then gently transferred to a 10 ml polypropylene column. The beads were washed with 10 column volumes of wash buffer (2 M NaCl, 20 mM Tris pH 6.8, 60 mM imidazole and 1% triton). hnRNP-K protein was eluted using 5 ml elution buffer (1 M imidazole 5% glycerol, pH 8.0). The hnRNP-K protein was dialysed against phosphate buffered saline. The purity and concentration were analysed using coomassie stained gels and absorbance at 280 nm respectively.

### 2.7 Site-directed mutagenesis

Tyrosine 458 of hnRNP-K was mutated to phenylalanine using the primers; forward 5′CAGTGTGAAGCAGTTTGCAGATGTTGAAG3′ and reverse 5′CTTCAACATCTGCAAACTGCTTCACACTG3′. Mutations were introduced using Quick-Change mutagenesis (Agilent Technologies).

### 2.8 In vitro transcription and biotinylation of UCP2 RNA

1 μg of plasmid containing full length UCP2 with 5′ and 3′ UTR’s (1–1647 nt) was linearized with Eco RV. The linearized plasmid was purified using acetate/ethanol precipitation. UCP2 was *in vitro* transcribed using MAXIscript *in vitro* transcription kit (Ambion). Transcription was confirmed by gel electrophoresis and concentration was determined using an absorbance at 260 nm. The *in vitro* transcribed RNA was biotinylated using the Pierce RNA 3′-biotinylation kit from Thermo Scientific according to the manufacturer's instructions.

### 2.9 Src-mediated phosphorylation of hnRNP-K

Purified hnRNP-K was phosphorylated by activated Src (Sigma). Briefly, 500 ng of purified hnRNP-K was incubated with 1 ng of active src in kinase assay buffer (5 mM MOPS, pH 7.2, 2.5 mM glycerol 2-phosphate, 4 mM MgCl2, 2.5 mM MnCl2, 1 mM EGTA, 0.4 mM EDTA, 50 ng/μl BSA, 50 μM DTT and 0.26 mM ATP) for 15 min at 30 °C.

### 2.10 UCP2 mRNA binding assay

300 ng of biotinylated UCP2 RNA was incubated with 500 ng of purified Src-phosphorylated or non-phosphorylated hnRNP-K in binding buffer (50 mM NaCl, 10 mM HEPES pH8.0, 0.1 mM EDTA and 1 mM EDTA) for 30 min in a volume of 30 μl at room temperature. The volume was then increased to 500 μl using binding buffer. Streptavidin beads were added to the mixture and incubated for 1 h on a roller at 4 °C. The beads were washed using binding buffer supplemented with 0.01% triton. Proteins were eluted using sample buffer and loaded onto a 7.5% gel and transferred onto a nitrocellulose membrane. Proteins were visualized using chemiluminescence.

### 2.11 Reactive oxygen species

ROS was detected in cells using the fluorescent marker dichlorfluorescein. Briefly, cells were placed in serum-free medium and activated with 100 ng/ml TNFα for 30 min with or without a 10 min preincubation of Ang1. Media was then replaced with serum-free media lacking phenol red and containing 10 μM 2′7′-dichlorfluorescein-diacetate (DCFH-DA) and incubated for 15 min. After washing twice with serum-free media lacking phenol red, fluorescence and phase contrast microscopy was used to image cells. For quantification the fraction of fluorescent cells counted in 5 fields for each treatment in each experiment was recorded.

## 3 Results

**Fig. 1:**
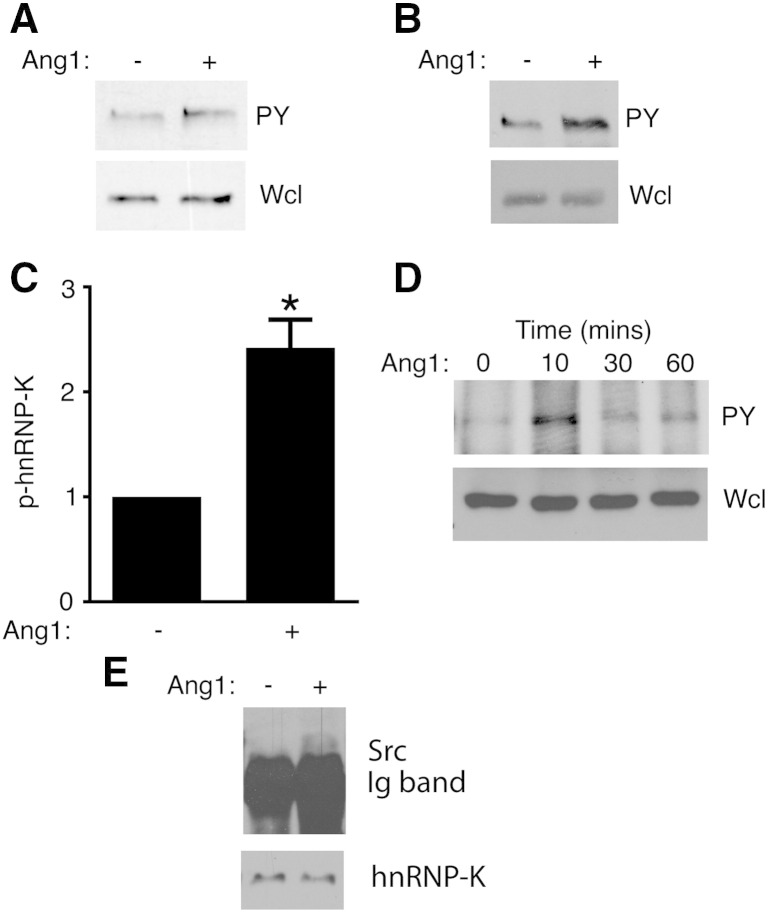
Ang1 activates phosphorylation of hnRNP-K and interaction with Src. Endothelial cells were challenged with Ang1 for 30 min. Lysates were immunoprecipitated with anti-phosphotyrosine. Immunoprecipitates (PY) and whole cell lysates (Wcl) were probed for hnRNP-K by immunoblotting. A, HUVEC and B, EA.hy926. C, hnRNP-K immunoprecipitated from EA.hy926 with anti-phosphotyrosine was quantified by densitometric scanning of blots. Data are means relative to control-treated cells (*p* < 0.05, Student's ‘*t*’ test). D, Time course of hnRNP-K phosphorylation in EA.hy926. E, EA.hy926 cells were stimulated with Ang1 for 10 min before lysis and immunoprecipitation of hnRNP-K. Immunoprecipitates were probed for co-immunoprecipitating Src as indicated.

The signal transduction pathways mediating the actions of Ang1 in endothelial cells are incompletely defined. To identify novel signalling intermediates involved in Ang1 action we performed anti-phosphotyrosine immunoprecipitations from endothelial cells treated with control vehicle or Ang1 and identified recovered proteins by mass spectrometry (data not shown). A protein consistently increased in the phosphotyrosine-containing fraction from Ang1-treated cells was the RNA binding protein hnRNP-K, suggesting that Ang1 can activate hnRNP-K phosphorylation. To confirm this directly, HUVEC were stimulated with Ang1 and anti-phosphotyrosine immunoprecipitates from these cells probed by immunoblotting with anti-hnRNP-K antibodies. This revealed increased hnRNP-K in the immunoprecipitates following Ang1 treatment (Fig. 1A). Similar results were obtained with the endothelial line EA.hy926 (Fig. 1B, C). Activation of phosphorylation occurred within 10 min of ligand addition (Fig. 1D). We note that hnRNP-K was among a group of proteins recently reported by others to become tyrosine phosphorylated in response to Ang1 [17].

The best-characterized mechanism for tyrosine phosphorylation of hnRNP-K is that mediated by Src. This tyrosine kinase can directly bind to hnRNP-K [18] leading to phosphorylation on a number of tyrosine residues in the RNA binding protein [19,20]. We were interested therefore to test whether Ang1 affects interaction of Src with hnRNP-K. Endothelial cells were activated with Ang1, hnRNP-K immunoprecipitated and immunoprecipitates probed for the presence of Src by immunoblotting. As shown in Fig. 1E, Ang1 stimulated Src interaction with hnRNP-K.

**Fig. 2:**
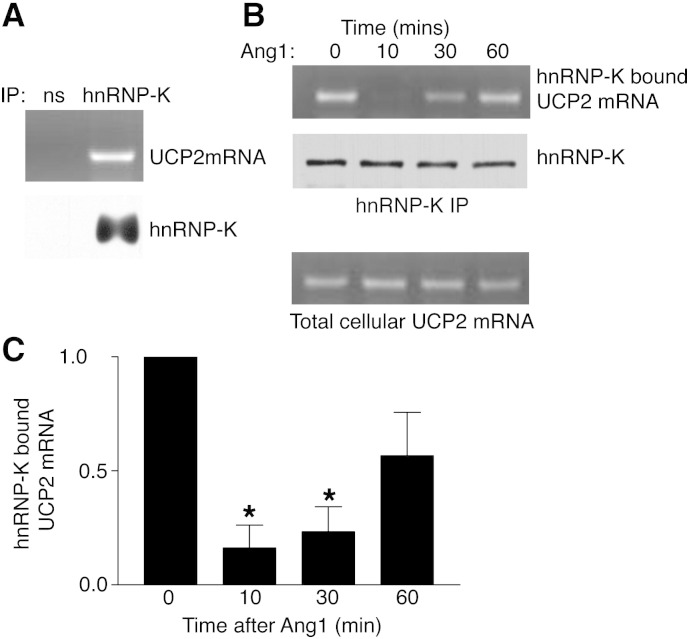
Ang1 stimulates release of UCP2 mRNA from hnRNP-K. A, EA.hy926 cell lysates were immunoprecipitated with non-specific antibody or anti-hnRNP-K and bound UCP2 mRNA detected by RT/PCR. B, Cells were treated with Ang1 for the times indicated. hnRNP-K was immunoprecipitated and UCP2 mRNA bound to hnRNP-K was detected by RT/PCR. C, UCP2 mRNA co-immunoprecipitated with hnRNP-K was quantified by scanning gels from 3 independent experiments. Data were normalized to hnRNP-K immunoprecipitated (*p < 0.05, Student's ‘*t*’ test).

hnRNP-K can bind to specific mRNA species, including transcripts encoding the mitochondrial protein UCP2 [21]. In order to determine the potential functional significance of Ang1-induced hnRNP-K phosphorylation we tested the effect of the ligand on the ability of hnRNP-K to bind UCP2 mRNA. hnRNP-K was immunoprecipitated from cell lysates and probed for bound UCP2 mRNA. UCP2 mRNA was found bound to hnRNP-K in endothelial cells under basal conditions (Fig. 2A). The effect of Ang1 on this was examined by stimulating cells with the ligand, lysing cells, immunoprecipitating hnRNP-K and probing for bound UCP2 mRNA. Surprisingly Ang1 stimulated a rapid decrease in UCP2 mRNA bound to hnRNP-K (Fig. 2B). Similar to activation of hnRNP-K phosphorylation, Ang1 activated UCP2 mRNA dissociation from hnRNP-K within 10 min (Fig. 2B,C).

**Fig. 3:**
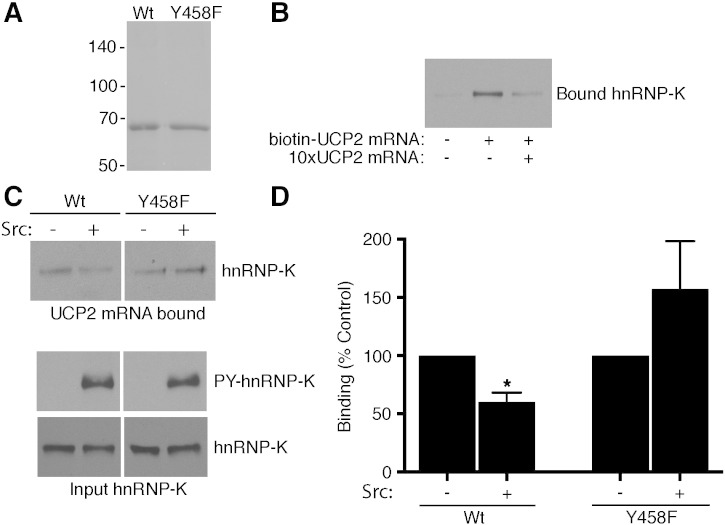
UCP2 mRNA binds purified hnRNP-K and is regulated by Src. A, Coomassie stained gel of recombinant His6-tagged wild-type hnRNP-K or Y458F hnRNP-K expressed in *E. coli* and purified on a nickel column. Positions of molecular mass markers are indicated in kDa. B, Binding of UCP2 mRNA and wild-type hnRNP-K *in vitro*. Recombinant hnRNP-K was incubated in the absence of UCP2 mRNA, with biotinylated UCP2 mRNA or with biotinylated UCP2 mRNA in the presence of a ten-fold excess of non-biotinylated UCP2 mRNA as indicated. Biotinylated mRNA was recovered with immobilized streptavidin and bound hnRNP-K detected by immunoblotting. C, Recombinant hnRNP-K or recombinant hnRNP-K in which tyrosine-458 was changed to phenylalanine (Y458F) was phosphorylated by addition of Src and then tested for binding to biotinylated UCP2 mRNA as above. Blots of bound hnRNP-K as well as input hnRNP-K and tyrosine phosphorylation of input hnRNP-K are shown as indicated. D, Binding of hnRNP-K to UCP2 mRNA was analysed in vitro as above and blots quantified by scanning. Binding is normalized to that in the absence of phosphorylation for each hnRNP-K and presented as means and SEM for three independent experiments (*p < 0.05, Student's ‘*t*’ test, for the effect of phosphorylation).

To examine further the interaction of UCP2 mRNA and hnRNP-K we expressed human hnRNP-K in *E. coli* and performed *in vitro* binding assays with the purified protein (Fig. 3). Incubation of biotinylated UCP2 mRNA with hnRNP-K followed by recovery of the mRNA using streptavidin beads confirmed direct binding between the transcripts and hnRNP-K *in vitro* (Fig. 3B). Our finding that Ang1 activates Src interaction with hnRNP-K and tyrosine phosphorylation prompted us to test the effects of Src-mediated phosphorylation of hnRNP-K on UCP2 mRNA binding *in vitro*. Incubation of purified hnRNP-K with Src resulted in tyrosine phosphorylation of the RNA binding protein (Fig. 3C), consistent with previous reports, and our findings in endothelial cells (Fig. 1). Importantly, UCP2 mRNA binding to hnRNP-K was decreased following tyrosine phosphorylation of hnRNP-K by Src (Fig. 3C). Several tyrosine residues in hnRNP-K are known to be phosphorylated by Src [20,22]. However, one residue in particular, Y458, has been shown to be critical for regulating binding with at least one mRNA species. Reticulocyte 15-lipoxygenase (LOX) mRNA binds hnRNP-K via a C/U-rich region in its 3′UTR and this binding is disrupted by Y458 phosphorylation [22,23]. To test whether Y458 phosphorylation is also involved in suppression of UCP2 mRNA interaction with hnRNP-K we constructed a mutant version of hnRNP-K with phenylalanine in place of tyrosine at this position. Comparison of the effects of Src-mediated phosphorylation on UCP2 mRNA binding between wild-type and Y458F versions of hnRNP-K revealed that in contrast to the wild-type protein, Src did not suppress UCP2 mRNA binding to the Y458F mutant (Fig. 3C,D). These data show that, as with LOX mRNA binding, UCP2 mRNA binding to hnRNP-K is inhibited by Y458 phosphorylation by Src.

**Fig. 4:**
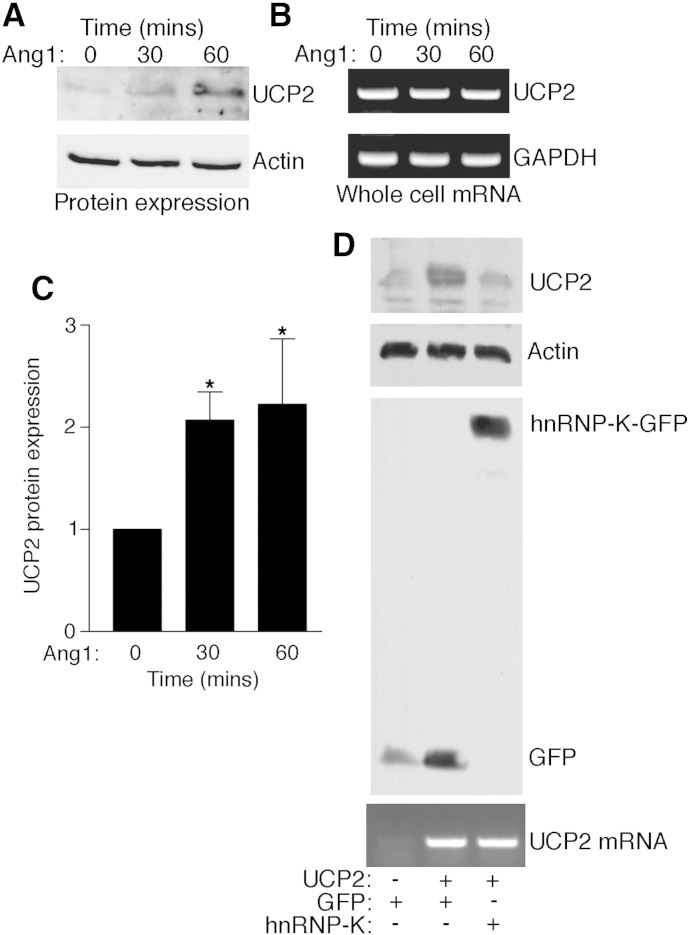
Ang1 activates UCP2 protein expression in endothelial cells. A, EA.hy926 cells were treated with Ang1 for the times indicated and UCP2 protein detected by immunoblotting. B, UCP2 and GAPDH mRNA were detected in cell lysates by RT/PCR following Ang1 activation. C, UCP2 protein was quantified by scanning blots from three independent experiments. UCP2 protein levels were normalized to actin and data are presented as mean and SE and shown relative to UCP2 in control-treated cells (*p < 0.05, Student's ‘*t*’ test). D, hnRNP-K suppresses UCP2 translation. CHO cells were transfected with plasmids containing nucleotides 1-1647 of UCP2 together with a plasmid encoding either hnRNP-K-GFP or GFP as indicated. Approximately 18 h post-transfection cells were lysed and UCP2 protein detected in cell lysates by immunoblotting. Blots were stripped and re-probed for GFP to confirm expression of hnRNP-K-GFP and GFP in appropriate cell lysates. mRNA was extracted from cell lysates and UCP2 mRNA detected by RT/PCR (15 cycles).

The effects of Ang1 on UCP2 mRNA suggest that the ligand may affect UCP2 protein levels. This was examined and Ang1 was found to stimulate a significant increase in UCP2 protein expression, without increasing total cellular UCP2 mRNA (Fig. 4A–C).

The ability of Ang1 to stimulate release of UCP2 mRNA from hnRNP-K and increase UCP2 protein expression without increasing cellular UCP2 mRNA levels suggests that the RNA-binding protein might act to hold UCP2 transcripts in a translationally inactive state that is relieved by Ang1-induced transcript release. To test whether hnRNP-K suppresses translation of UCP2, therefore, cells were transfected with a plasmid encoding UCP2, including the 5′ and 3′ untranslated regions, together with a plasmid encoding GFP (control) or GFP-tagged hnRNP-K. Levels of expression of transfected UCP2 were too low to detect when endothelial cells were transfected (data not shown) so these experiments were performed with CHO cells. As shown in Fig. 4D, cells transfected with UCP-2 plasmid along with GFP demonstrated increased expression of UCP2 protein as expected. However, co-transfection with hnRNP-K decreased expression of UCP2 protein. Furthermore, cells transfected with a plasmid encoding UCP2 showed, as expected, increased cellular UCP2 mRNA levels but, in contrast to the effects of hnRNP-K expression on UCP2 protein, UCP2 mRNA levels were not decreased by hnRNP-K (Fig. 4D). These data indicate that hnRNP-K can act to suppress UCP2 translation.

**Fig. 5:**
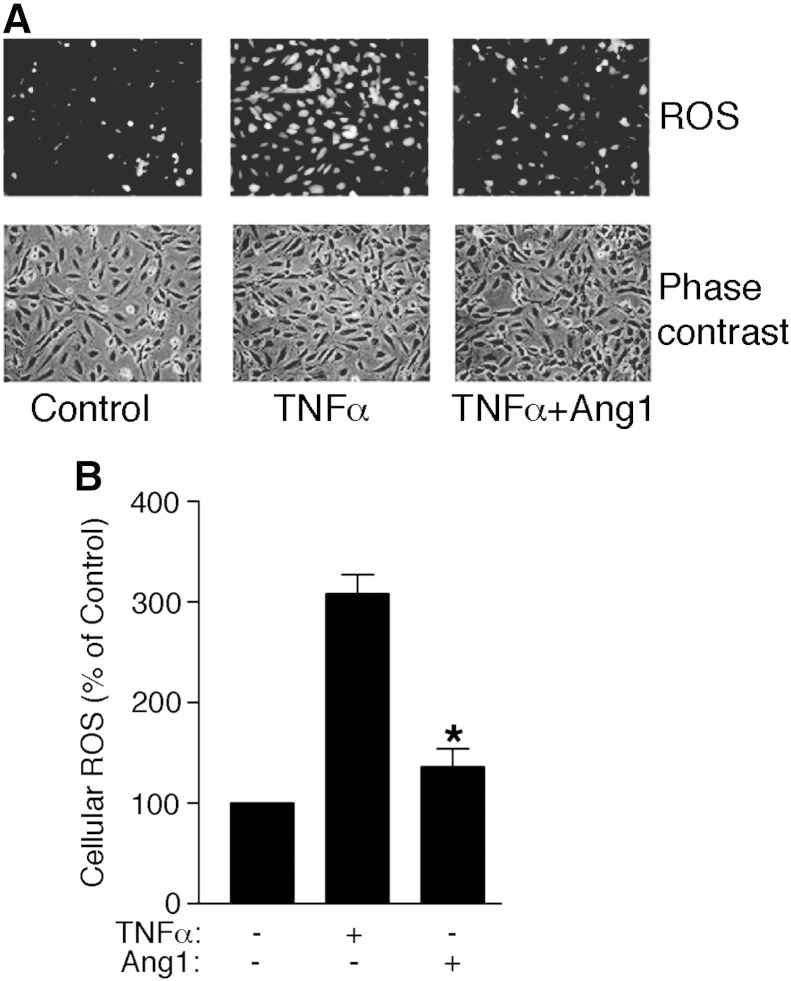
Ang1 inhibition of ROS generation. Endothelial cells were stimulated with TNFα in the absence and presence of Ang1 before detection of ROS using DCFH-DA. Data are presented as means and SE and shown relative to the fraction of ROS-positive cells in control-treated cells (*p < 0.05, for Ang1 inhibition of TNF-activated ROS, Student's ‘*t*’, test *n* = 3).

UCP2 is known to affect cellular reactive oxygen species (ROS) and the protein inhibits ROS production from the mitochondria [24,25]. Upregulation of UCP2 protein by Ang1, therefore, would be expected to have a suppressive effect on ROS production. To gain insight into the possible biological significance of Ang1 regulation of UCP2 protein we examined whether Ang1 could modify ROS production. TNF-α has previously been reported to stimulate mitochondrial ROS production in endothelial cells [26]. We found that TNF-α-treated endothelial cells demonstrated increased ROS production (Fig. 5). Pre-treating cells with Ang1 caused a significant inhibition of TNF-α-induced Ros production (Fig. 5).

## 4 Discussion

The present study identifies a new mechanism by which Ang1 regulates UCP2, a mitochondrial protein important in regulating ROS levels in cells. We find that in endothelial cells UCP2 mRNA is bound to the RNA binding protein hnRNP-K. Ang1 stimulates interaction of the tyrosine kinase Src with hnRNP-K and tyrosine phosphorylation of the RNA-binding protein. This is associated with the release of UCP2 mRNA and upregulation of UCP2 protein in the cells. This mechanism provides a pool of UCP2 mRNA ready for translation, allowing Ang1 to rapidly upregulate UCP2 protein without the need to activate gene expression. The increased speed afforded by such a post-transcriptional response may be particularly important for UCP2 as this protein has a very short half-life [27] and regulates levels of an intermediate, ROS, whose actions can be potentially damaging to the cell.

hnRNP-K was originally identified as part of the heterogenous ribonucleoprotein complexes involved in processing pre-mRNA [28]. The protein is localized primarily to the nucleus but is also found in lower abundance in the cytoplasm [29–32]. hnRNP-K has roles in control of gene expression by modulating chromatin remodeling, transcriptional control and splicing, as well as transporting specific transcripts into the cytosol and regulating mRNA translation by binding transcripts [33]. The ability of hnRNP-K to regulate mRNA translation appears to be important for control of local protein expression in cells. This was shown recently in oligodendrocytes where hnRNP-K was found to bind mRNA encoding myelin basic protein, holding these transcripts in a translationally silent state whilst transporting them to the membrane and areas of myelination [34]. In these cells integrin activation resulted in hnRNP-K tyrosine phosphorylation releasing the transcripts and leading to localized increased myelin basic protein translation [34].

The interaction of specific mRNA with hnRNP-K is regulated by phosphorylation state of the RNA-binding protein, with increased tyrosine phosphorylation leading to release of specific mRNAs from the protein [20,35]. Conversely, there are instances of enhanced binding of some transcripts in response to tyrosine phosphorylation of the protein, for example platelet-derived growth factor enhances phosphorylation of hnRNP-K and its binding to mRNA encoding myosin regulatory light-chain interacting protein [36]. This suggests that tyrosine phosphorylation-dependent regulation of hnRNP-K interaction with transcripts is complex, transcript specific and is likely to involve other proteins.

The best-described example of signal-regulated mRNA interaction involving phosphorylation of hnRNP-K is in the control of reticulocyte LOX. Expression of LOX protein is suppressed in immature erythroid cells by binding of LOX mRNA to hnRNP-K which prevents its translation [37]. Phosphorylation of hnRNP-K via Src causes release of LOX mRNA and upregulation of LOX protein [20]. LOX mRNA binding to hnRNP-K is mediated by C/U rich elements in the 3’ untranslated region [23]. Similar elements are present in the 3′ region of UCP2 mRNA. This, together with our finding that, like LOX mRNA, UCP2 mRNA binding to hnRNP-K is sensitive to phosphorylation status of Y458, suggests that both mRNA species may share a similar mechanism of phosphorylation-induced release. hnRNP-K contains 17 potential tyrosine phosphorylation sites and it is likely that different agonists activate phosphorylation of different tyrosines leading to different effects on mRNA binding. Indeed, insulin has been reported to stimulate tyrosine phosphorylation of hnRNP-K and, in an insulin receptor overexpressing cell line, this increased UCP2 mRNA binding [21]. The mechanisms responsible for the differing effects of Ang1 and insulin on hnRNP-K:mRNA binding are not known but may reflect differences in phosphorylation sites stimulated by the two ligands and/or differences between cell types.

Uncoupling protein-2 is a 33 kDa anion carrier found in the mitochondrial inner membrane [38]. The protein is important for control of mitochondrial membrane potential [39] and generation of reactive oxygen species [40]. Increased UCP2 is associated with partial depolarization of the mitochondrial membrane and suppression of mitochondrial ROS production [25]. UCP2 mRNA levels in tissues have been reported as generally high whereas protein levels are low, suggesting that translation of the protein is tightly controlled [41]. Consistent with this, starvation and lipopolysaccharide induce substantial changes in UCP2 protein levels *in vivo* without accompanying effects on mRNA levels [41]. It has also been reported that UCP2 protein has a short half-life of around 30 min [27]. Translational control of UCP2, together with the short half-life of the protein, may be important for allowing rapid responses in intracellular UCP2 protein levels to cellular stimuli.

A key function of UCP2 to emerge in recent years is its ability to suppress mitochondrial generation of ROS [24,25]. Elevated ROS can have detrimental effects on cells and tissues, for example increased ROS has been associated with enhanced expression of endothelial adhesion molecules and endothelial apoptosis as well as vascular dysfunction and increased atherosclerosis [42,43]. Furthermore mitochondrial DNA is a critical target for oxidative damage because of their proximity to electron chain and the crucial roles played by mitochondrial DNA products. It is clearly important therefore that levels of ROS are tightly controlled. UCP2 has been shown to protect endothelial cells via its ability to inhibit mitochondrial ROS generation, with increased expression of UCP2 protein inhibiting ROS-mediated apoptosis in endothelium [25]. Overexpression of UCP2 also suppresses endothelial NFkB activation and enhances vascular relaxation [25]. Consistent with a protective role of UCP2 in the vasculature, transgenic low density lipoprotein receptor deficient mice lacking UCP2 in circulating cells exhibit increased atherosclerosis [44]. Similarly, UCP2-deficient mice display elevated atherosclerosis, decreased endothelial nitric oxide levels and increased vascular adhesion molecule expression [45] as well as increased salt-induced hypertensive changes [46]. Thus UCP2 appears to have a broadly protective effect in endothelium and the vasculature. Ang1 is also known to have vascular protective effects and suppresses inflammatory gene expression in endothelial cells and blocks microvessel regression, vascular leakage and inflammation in vivo [6]. Induction of UCP2 protein by Ang1 therefore could be considered an additional protective effect of Ang1, to limit ROS levels in endothelium.

In conclusion, our data shows that Ang1 activates tyrosine phosphorylation of hnRNP-K, release of UCP2 mRNA from the RNA-binding protein and increasing UCP2 protein levels in the cells. This increase in UCP2 is likely to be important in preventing excess ROS production. Significantly, this post-transcriptional control mechanism will allow rapid responses to the ligand.
